# A survey of clinical empathy training at UK medical schools

**DOI:** 10.1186/s12909-022-03993-5

**Published:** 2023-01-19

**Authors:** Rachel Winter, Andy Ward, Robert I Norman, Jeremy Howick

**Affiliations:** 1 Stoneygate Centre for Empathic Healthcare, Leicester Medical School, George Davies Centre, University of Leicester, University Road, LE1 7RH Leicester, England; 2 Leicester Medical School, College of Life Sciences University of Leicester, George Davies Centre, LE1 7RH Leicester, England; 3 The Oxford Empathy Programme, Faculty of Philosophy, University of Oxford, OX2 6GG Oxford, England

**Keywords:** Clinical empathy, Medical education, Survey

## Abstract

**Background:**

The benefits of enhancing practitioner empathy include better patient outcomes and improved job satisfaction for practitioners. Evidence suggests empathy can be taught and empathy is listed as an outcome for graduates in the General Medical Council requirements. Despite this, empathy training is not mandatory on medical school curricula and the extent to which medical students are given empathy-specific training is unknown.

**Aim:**

To conduct a survey of empathy training currently offered to medical students in UK medical schools.

**Methods:**

An invitation to participate in an online survey was sent to all UK medical schools (*n* = 40). The survey was developed through a consultancy and pilot process to ensure validity and reliability. Questions explored what empathy-focused training is offered, and asked educators whether or not they believed that current provision of empathy training is sufficient. In parallel, medical school websites were searched to identify what information regarding empathy-focused training is described as being part of the degree course. Descriptive statistics were used to describe empathy training delivery from the results of the online materials survey and closed survey questions. Thematic analysis was used to explore free text comments.

**Results:**

Response rate was 70% (28/40), with 28 medical schools included in the analysis. Twenty-six schools reported that their undergraduate curriculum included some form of empathy-focused training with variation in what, when and how this is delivered. Thematic analysis revealed two overarching themes with associated sub-themes: (i) empathy-focused training and development (considering where, when and how empathy training should be integrated); (ii) challenges presented by including empathy on the curriculum (considering the system, students and faculty). All schools agreed empathy training should be on the undergraduate curriculum.

**Conclusion:**

This is the first nationwide survey of empathy-focused training at UK medical schools. While some form of empathy-focused training appears to be provided on the undergraduate curriculum at most UK medical schools, empathy is rarely specifically assessed. Most medical educators do not feel their school does enough to promote empathy and the majority would like to offer more.

**Supplementary Information:**

The online version contains supplementary material available at 10.1186/s12909-022-03993-5.

## Background

Empathic healthcare can improve patient outcomes, [1–3] enhance the quality of patient care, [4] augment practitioner performance, [5] and reduce practitioner burnout [6]. The General Medical Council requires newly qualified doctors to be able to demonstrate empathy and compassion to patients, [7] and there is a growing recognition that training and assessment in clinical empathy should be included on the undergraduate curriculum [8]. Empathy can be fostered in medical students and professionals through training [9–11] .

The extent to which empathy-specific training is offered in UK medical schools is unknown and there is no standard empathy training available. In parallel, there is evidence too that empathy may decline during medical school [12]. This survey of medical educators and medical school websites seeks to establish the degree to which empathy training is routinely included in medical education within UK medical schools.

There are many accepted definitions of empathy in the clinical setting [13, 14]. In addition, there is overlap between clinical empathy and other related but different concepts, such as interpersonal and communication skills. We are following what appears to be an emerging consensus [5–18] that therapeutic empathy is the ability to understand the patient’s perspective, to communicate this understanding, and to act on it in a helpful (therapeutic) way [19].

In this survey, we asked the research question: to what extent is empathy training currently included in the undergraduate medical curriculum across UK medical schools?

### Aims and objectives

Our study aims are to: (1) determine whether empathy-focused training is offered to UK undergraduate medical students; (2) identify what empathy-focused training is offered to UK undergraduate medical students; (3) to explore medical educators perceptions of introducing empathy to the medical school curriculum.

## Methods

Our study objectives are to: 1) conduct a survey of representatives of all UK undergraduate medical schools to determine whether and what empathy-focused training is offered as part of their curriculum and whether there is an appetite for more; 2) conduct a survey of all UK undergraduate medical school websites to determine what empathy-focused training is offered as part of their curriculum.

We recognise that many teaching activities, for example communication skills training, can develop empathy to some degree. To capture these activities that are related to empathy but not labelled as empathy, we define ‘empathy-focused training’ as any educational activity that has been developed with the primary outcome of fostering clinical empathy.

### Study design

This is a cross-sectional study of UK medical schools and medical educators. The Consensus-Based Checklist for Reporting Survey Studies (CROSS) [20] has been adopted to report our findings. The survey protocol is registered with Open Science Framework [21].

### Survey design

The lack of a standardised survey questionnaire that addressed the aims of this study led to the development of our own (see Additional file 1). The survey was generated on Jisc Online Survey software. Questions were based on a systematic search of studies and reviews exploring empathy training and curricula at medical schools, [9–11, 22] and through a review of related study-specific questionnaires of teaching (including communication skills (not explicitly empathy-focused)) at UK medical schools [23–25]. A series of closed questions plus some open-ended questions inviting free text responses were used. Questions fell in to four domains: (a) questions about the institution and role of respondent, (b) questions about empathy-focused training, (c) questions about the assessment of empathy and (d) questions about the respondents’ opinions of empathy-focused training. Survey questions were put through a consultation process with a group of medical educators, clinicians and curriculum developers (*n* = 8) to ensure face validity and that questions were clear and unambiguous. The survey was piloted with senior medical educators from three different medical schools.

### Sample characteristics

Medical education leads (MELs) at forty-one medical schools offering a standard entry and/or graduate entry accredited medical degree for national students were contacted through the Medical Schools Council to take part in this study.

### Survey administration

MELs were sent a description of the study (see Additional file 2) with contact details for the primary investigator and a link to the survey platform, and asked to nominate a representative with knowledge of the curriculum content. A follow-up reminder was sent to medical schools who had not responded at 10 and 17 days. For medical schools who had not completed the survey following the second reminder, alternative contacts at the medical school were emailed to request participation.

In parallel, an online survey of UK medical school websites and official online materials (prospectuses/course pages) was conducted by RW between 2 February 2022 and 7 March 2022. University and medical school websites, along with, where available, online prospectuses and programme specifications, were manually searched for written, audio and visual course and curricula information relating specifically to; the current provision of specific empathy-focused training; the current provision of communication/interpersonal skills training; empathy as a skill or attribute that is assessed for in relation to selection to medical school. In addition, the terms ‘empathy’, ‘empathic’, ‘empathetic’ and ‘compassion’ were searched for using the internal search engine on each university website with all results viewed to ensure that any information about empathy-focused training/teaching activities was captured. In addition, each university website was searched for the medical schools’ ‘programme specification’. If not found, a search using Google was run (using the name of the medical school and ‘programme specification’). Where a programme specification was identified, this was searched to identify details relating to empathy-focused training and learning outcomes. The types of course offered by each medical school (standard entry, graduate entry, medicine with foundation or gateway year) was also recorded.

### Data analysis

Responses from medical educators were anonymised and assigned a code. Descriptive statistics were used to summarise data collected from closed survey questions to help identify any meaningful trends. Thematic analysis, to allow for themes to emerge from the data rather than be driven by an existing framework, [26] was used to explore data collected from open survey questions. Themes were identified by RW using a Word document to organise data with supporting quotes identified for each theme. A second author (JH) reviewed themes and meanings, with any disagreement resolved through discussion. RW and JH discussed and selected quotes for inclusion within the main study.

## Results

We sent surveys to MELs at 41 schools. One medical school declined to take part as they are a new medical school that had not yet completed their first year intake of students and we did not include it. Twenty-eight medical schools (70%) completed the survey. England, Scotland and Wales were all represented within the schools that responded. Results are summarised in Table 1.

**Table 1:** Summary of survey findings

	**No. of responses**	**%**
**Does your curriculum include formal empathy-focused training?**		
Yes	26	93
No	1	4
Unsure	1	4
**Is there a dedicated empathy-focused programme or module or are activities integrated into other modules?**		
Dedicated empathy-focused programme or module	2	7
Empathy-focused activities are integrated	16	57
Both	7	25
Unsure	1	4
**Have specific empathy-focused intended learning outcomes been developed associated with this?**		
Yes	17	61
No	5	18
Unsure	3	11
**When does specific empathy-focused training take place?**		
Foundation or Gateway Year (Year 0)	4	14
Year 1	23	82
Year 2	19	68
Year 3	15	54
Year 4	13	43
Year 5	14	50
Other	2	7
**Are empathy-focused training activities part of the compulsory or optional curriculum?**		
Compulsory curriculum	21	75
Optional	0	0
Both	5	18
Unsure	1	4
**What teaching methods are employed to deliver empathy-focused teaching activities?**		
Lectures	10	36
Problem-based learning	1	4
Seminars	5	18
Small group work	26	93
Online activities	6	21
Clinical experience	18	64
Other	6	21
**Who is responsible for the delivery of empathy-focused training?**		
Clinical academics	23	82
Academics	11	39
NHS clinicians	21	75
Clinical teaching fellows	17	61
Patients	11	39
Other (including simulated patients and students)	3	11
**Does your medical school provide any form of training or development for faculty and clinical educators around clinical empathy and teaching this to students?**		
Yes	12	43
No	8	29
Unsure	8	29
**Is there anything that fits our definition of clinical empathy, which you feel your curriculum delivers, but is labelled as something else?**		
Yes	19	68
No	6	21
Unsure	3	11
**If empathy-focused training is offered, is it evaluated?**		
Yes	18	64
No	4	14
Unsure	3	11
**If empathy-focused training is offered, is student feedback sought?**		
Yes	18	64
No	2	7
Unsure	5	18
**Is student empathy assessed at any point during the degree programme?**		
Yes	22	79
No	1	4
Unsure	4	14
**If student empathy is assessed, how is it assessed? (select all that apply)**		
Self-assessment	3	11
Reflective practice	15	54
Written exam	1	4
OSCE	24	86
Portfolio activities	14	50
Other	4	14
**Are any empathy-specific tools used to measure student empathy? (select all that apply)**		
None	22	79
Barrett-Lennard Relationship Inventory-empathy understanding (BLRI)	0	0
Consultation and Relational Empathy (CARE) measure	0	0
Empathy Construct Rating Scale (ECRS)	0	0
Empathy Quotient (EQ)	0	0
Jefferson Scale of Empathy (JSE)	1	4
Medical Condition Regard Scale (MCRS)	0	0
Reynolds Empathy Scale (RES)	0	0
Therapist Empathy Scale (TES)	0	0
Toronto Empathy Questionnaire (TEQ)	0	0
Other	2	7
**Is the assessment of empathy, or a student’s ability to empathise considered in the admissions to medical school process?**		
Yes	19	68
No	1	4
Unsure/unable to comment	8	29
**Do you think clinical empathy should be taught at medical school?**		
Yes	27	96
No	0	0
**Do you think your medical school does enough to foster empathy in medical students?**		
Yes	7	25
No	12	43
Unsure	9	32
**Would you like to see more empathy-focused training on your undergraduate curriculum**		
Yes	23	82
No	4	14

Of the schools that responded, 14 (50%) offered only standard entry (undergraduate) medicine course, two (7%) offered only graduate entry medicine and 12 (43%) offered both. Eleven medical schools offered medicine with foundation or gateway year entry. Eleven (39%) reported that their curriculum is taught at another medical school. Of those completing the survey on behalf of their medical school, 24 (86%) described their role as being involved in both ‘curriculum design and delivery’. One described their role as ‘curriculum design’, and two reported their role as ‘other’ (and described their role as ‘Professionalism Lead’).

Eleven medical schools (27%) did not respond to requests to complete the survey. Of these, nine (82%) offered standard (undergraduate) medicine course, and two (18%) offered both standard entry and graduate entry medicine. Six schools (55%) offered medicine with foundation or gateway year entry. Schools who did not respond were located across England, Scotland and Northern Ireland.

### Main findings

To reflect our study aims, findings are presented under three main headings (see below).

1. Empathy-focused training.

- Whether empathy-focused training is included on the medical school curriculum.
- What empathy-training is included on the medical school curriculum.

2. Evaluation and assessment.

- If and how empathy-focused training is evaluated, or student empathy assessed.

3. Future development.

- Whether there is a requirement for more empathy-focused training to be included on the medical school curriculum.

### Empathy-focused training

Twenty-six (93%) medical schools reported that their curriculum included some form of formal empathy-focused training or educational activity designed to foster empathy. Of these, eight (29%) stated there was a dedicated empathy-focused programme or module as well as empathy-focused training activities being integrated into other courses or modules. Sixteen (57%) reported empathy-focused training activities were integrated into other courses or modules. Two medical school stated there was an empathy-focused programme or module only. One school stated there was no formal empathy-focused training and one was unsure. All of the schools that reported some form of empathy-focused training was offered to students indicated this was done so as part of the mandatory curriculum.

Specific empathy-focused training was reported to happen most frequently in the first and second year of medical school. Twenty-one (75%) medical schools reported empathy-focused training as mandatory, whilst 5 (18%) reported it was included on mandatory and optional curriculums.

There was a wide variety in the way empathy training is reported to be delivered by schools. Of those who report that empathy training is offered, most report this to be through lectures (35%), small group work (93%) and clinical experiences (64%). Clinical academics (82%), NHS clinicians (75%) and clinical teaching fellows (61%) were most frequently reported to delivery empathy-focused training. Eleven medical schools (39%) reported that patients assist with the delivery of empathy training.

Twelve medical schools (43%) reported some form of training or development for faculty on clinical empathy was delivered to those responsible for teaching this to students. Eight schools (29%) were unsure if any training was provided and eight (29%) reported no training was offered. Of the responders who provided examples of training given, these included: ‘clinical tutor training days’ with empathy being discussed as part of the whole communication delivery; tutor training on the ‘model of empathy’; training workshops on ‘effective consulting’; an e-learning package on clinical empathy as part of initial training to work with students.

Nineteen schools (68%) reported their curriculum delivered additional teaching that they felt fitted our definition of empathy, but was labelled as something other than empathy-focused training. Examples included communication skills training; patient experiences; patient-centred care; professionalism.

### Evaluation and assessment

Of the 26 (93%) medical schools that reported their curriculum did include some form of formal empathy-focused training, 18 (64%) reported that training was evaluated and that student feedback on training was sought. Seventeen (61%) reported specific empathy-focused intended learning outcomes (ILOs) had been developed associated with training.

Twenty-two (79%) of schools reported student empathy is assessed at some point during the degree programme, with the most (86%) stating assessment was through Observed Structured Clinical Examinations (OSCEs) and/or reflective practice (54%). Only one (4%) medical school reported the use of empathy-specific tools to measure empathy (the Jefferson Scale of Empathy – Student Version). One school reported that ‘empathy mapping’ was used to specifically assess empathy and one reported the Generic Consultation Skills (GeCoS) tool was used.

Nineteen medical schools (69%) stated a prospective medical student’s ability to empathise was assessed during the admissions process. Of these, five (18%) provided examples of how empathy was assessed, with all five stating through Multiple Mini Interviews or role-play scenarios. Eight schools (29%) reported they were unsure of, or unable to comment on admissions processes.

### Future development

Of the schools taking part in the survey, 96% felt clinical empathy should be taught at medical school. Seven (25%) participants felt their medical school does enough to foster empathy in students, however, twenty-three schools (82%) reported they would like to see more empathy-focused training on the undergraduate curriculum.

#### Thematic analysis

Responders were asked to:

- Describe in their own words what empathy-focused training their medical school provided.
- Describe whether their curriculum included teaching that could be considered to fit our definition of empathy [19] but labelled as something else.
- Give their opinion on how clinical empathy could be taught or how teaching could be improve.

Participant responses fell into two overarching themes: empathy-focused training and development, with sub-themes around where on the curriculum training is or should be included and how it is (or is assumed to be) integrated into other activities; and challenges presented in putting empathy on the curriculum (related to the system, the students or the faculty). A summary of themes and supporting quotes is provided in Table 2.

**Table 2:** Summary of themes and supporting quotes

Theme	Quotes
Empathy-focused training and development	
Empathy-training is delivered through dedicated teaching	We have developed a 2 week Massive Open Online Course on the platform Future Learn, which all students complete in either Year 1 of MBBS4 or Year 2 of MBBS5 as part of their curriculum. The learning objectives are assessed in OSCEs and all students are required to write a short reflection on their experience/learning from the MOOC. (MEL 1) The course "Developing Clinical Empathy" aims to help students develop an empathic practice that is personal and attuned to their patients. They learn about what empathy is & it's different facets, they consider their own experiences of empathy and reflect on their and they explore verbal and non-verbal cues with the aim of understanding key opportunities for showing empathy in whilst caring clinically for patients. (MEL 1) in years 1 & 2 small group workshops (preceded by a lecture) that focus on understanding the patient perspective, and experience and communication skills that communicate this to (simulated) patients and look at the therapeutic nature of the consultation. (MEL 2) There is a 1 h online workbook that outlines why we should be empathetic to our patients, how it differs from sympathy & reassurance, a model of empathy, and several activities designed to encourage students to identify empathetic responses to patient statements… (MEL 5) There is a clinical empathy programme that runs throughout the foundation year. (MEL 3) We have introduced longitudinal placements combined with a patient panel for the whole of year 4 specifically with the aim of students developing long term relationships with doctors and particularly patients to build their empathy. (MEL 10) we are redeveloping the last few months of our 5th year and I would like to see this [empathy] become a focus within that time (as well as in the earlier years of the course that we already have) (MEL 7) Specific simulated patient encounters to deal with empathy (MEL 16) initial small group experiential sessions focusing specifically on what empathy is and ways of communicating and acting empathically… (MEL 14)
Empathy-training is integrated into other teaching activities	teaching about empathy as part of clinical communication teaching activities: either about 'general' aspects of clinical communication (such as patient-centred aims of the consultation) or in specific situations (e.g. difficult communication/breaking bad news). (MEL 19) we discuss empathy and the use of empathetic statements routinely as part of our small group communication skills training. (MEL 20) Role play with actors focuses strongly on the students' ability to empathise. (MEL 20) In early years (1 and 2) we speak explicitly about empathy (what is it, how do we demonstrate it, what is the impact on the patient and the consultation) as part of an introduction to communication skills teaching. (MEL 4) All of the [communication] skills training includes discussion around empathy. (MEL 8) We have a Social Accountability (Community based placement) where all third year students choose a 4 week placement in local charities/schools/organisations (in areas of higher deprivation) and further development of empathy is a key feature of students reflections. (MEL 8) Empathy is discussed from the start of Y1 Communication skills—in the Introductory Lecture, where it is defined MEL 13 Students begin with triad role-play and the techniques for expressing empathy are practised and evaluated within basic clinical scenarios. Midway through Y1 and throughout Y2, Small group communication skills workshops include trained medical role-play actors. MEL 13 Small group experiential communication skills sessions—all sessions (6 sessions in years 4,5,6) include appreciating patient perspective and empathic responding (MEL 22) Clinical communication skills teaching sessions—simulated consultations in a wide range of contexts—using volunteer patients and actors. Empathy, patient centredness, and 'what matters to you' embedded in all sessions. (MEL 24) In the Final year, students attended small group teaching with role play on "Challenging Scenarios" and a full day on 'Breaking Bad News". Empathy focused training plays a very important role in these sessions as we refer to models of communication in which empathy is a key skills to use and demonstrate effectively. (MEL 1) aims and objectives are also threaded throughout the MBBS programme and each small group experiential session that students have includes the objective to "Practise communicating empathy using appropriate verbal and non-verbal skills" (MEL 1) In Year 3 this forms part of the GP day each fortnight where students step into role as patients to enhance their empathy (MEL 2) There is a compassionate holistic diagnostic detective module that includes elements of empathy and compassion training during years 1 and 2 (MEL 3) Attending to patient perspective with our patient partner interactions in communication skills teaching in all years (MEL 21) Medical humanities SSC y3 which has a range of options to consider empathy and develop this for patients and self-care too. (MEL 21) Communication Skills are taught from the first few weeks of Year 1. They are practised weekly by each student with simulated patients. Over the course of 8 sessions, they build up their skill. Throughout, the importance of seeking to understand the patient's perspective is emphasised for being as important as the biomedical aspects…They learn how to demonstrate empathy…This is all part of their first semester training in Consultation Skills (MEL 23) I would like to treat it as a spiral topic, to be revisited several times to deepen the student's ability to show empathy—rather than a one and done session. (MEL 5) We have a case based curriculum and one case in year one has a specific focus with learning outcomes around the importance of, and demonstrating empathy. (MEL 10) Empathic communication is integrated into pretty much all of the communication training, which occurs throughout the course. (MEL 14) activities where empathy is referred to include the Elective introduction (the communication lead is also the elective lead) and in 1–1 coaching for students referred as having specific identified challenges with this competency. Empathy also comes up as part of case based discussion on the ethics module – e.g. end if life. (MEL 6) Classroom sessions on empathy are only a modest start point—it is the integration of theory into the day to day workplace that matters. (MEL 6) It should be integrated in all teaching rather than specific module (MEL 27) In the early years, we have a lecture delivered by a patient representative on Compassion and this is very much linked to our empathy-focused training but the lead for this session calls it Compassion. (MEL 1) Personally, I think it needs some of its own space (so students get a handle on specific skills) but also must be fundamentally interwoven with the values and communication behaviours related to patient-centred care—which must be prevalent throughout the whole medical curriculum. (MEL 19)
Empathy is intrinsically taught through other activities	I think it is very difficult to untangle whether the primary aim of a teaching session is to improve empathy – it is taught with other skill and where the emphasis lies depend on who is teaching. (MEL 9) so I would say we have dedicated training on wellbeing (which helps awareness of emotional labour of care, suffering and compassion), self-awareness (bias, reaction to situations and triggers, choosing response rather than reaction), trauma-aware care, and compassion—all of which contribute to empathy, but we don't specifically badge it as 'empathy training' (MEL 7) Communication skills teaching for system focused patient consultation and simulation of the acutely ill patient (MEL 11) There is a mindfulness programme during year 1. (MEL 3) We have a large cohort of Patients as Educators (in excess of 800) and they work with students throughout the 5 years (teaching and assessment) to understand the impact of illness. (MEL 8) We do not use the term empathy-focused training but rather emphasise the importance of empathy in patient encounters (MEL 12) we don't label anything specifically as 'empathy training' but we include the above [definition of empathy as used by survey] which contribute to this construct (MEL 7) [Empathy is] taught to med students through the clinical skills teaching (MEL 16) this [empathy training] is woven throughout the curriculum and is consolidated through repetition across contexts. (MEL 13) I think that clinical empathy and associated strategies are predominately learnt via role models in practice (the hidden curriculum) (MEL 26)
Assumption that empathy training is included elsewhere	It is a large part of our communication course. It is also a focus of our patient delivered curriculum in neurology and women's health (MEL 9) We incorporate this into our communication skills offering (MEL 12) All clinical placements should have inbuilt opportunities to further develop clinical empathy, but I am unsure how much this occurs. (MEL 6) We have Community Attachment Scheme in Phase 1 (yr1) which focuses on the psychosocial impact of illness. (MEL 8) I suspect this is provided on an ad hoc basis in a variety of specialties. Is informally being delivered throughout the curriculum. (MEL 17) In later years empathic communication is integrated into all aspects of the communication sessions, whether explicitly badged as comms or as part of other areas—sexual health course for instance. (MEL 14) Session in week 1 designed and led by patient group using drama and interactive activities. Regular "Person of the week" throughout year 1 & term 1 of year 2. Interaction with person, students ask questions live. Early clinical exposure in community settings getting to know 3 people (child, adult, older adult) and presenting a holistic picture of their lives and care experiences. (MEL 18) We don't specifically say "empathy based training" as we are not teaching students to 'learn to sound empathic' as an isolated skill, or thinking about 'one item'. It is—hopefully—integrated. (MEL 6) There is specific reference to empathy in the Year 1 plenary, and it is almost always discussed in de-brief of SP sessions cross years/programmes. There is more work to be done, as emphasis may well vary by tutor. (MEL 6) Consultation skills sessions: Active listening, Dealing with difficult consultations, Elements of GI/GU history-taking (as per the UKCCC curriculum wheel including two sets of ILOs in one session), Breaking bad news (in Giving information), Talking with relatives, Shared decision-making, End of Life care (comms skills) (MEL 15) it seems this will come up in a number of ways, where teaching is small group and considering ethics, shared decision making, patient centredness, etc. The overall badge is "Clinical Communication and Professionalisation". The challenge is to make the importance of empathy more explicit to students. (MEL 6) I think that forms part of our communication skills programme due to the important impact of this on interacting with patients. (MEL 4) none of our course is labelled 'clinical empathy' but I think a lot is delivered that would fit that description (MEL 9) I would put NHS clinicians in [teaching empathy] but I'm not sure if they really focus on this but more on the clinical—depends on the clinician (MEL 25)
Challenges presented in putting empathy on the curriculum	
System-based challenges	Large cohorts are sometimes a barrier, as developing empathy can need specific individual work. It relates to the student's individual lens, world view, maturity and experience. And can need intensive 1–1 support where affiliated to, eg, an attitude/values weakness or an specific learning difficulty. (MEL 6) Scale is a challenge to standardisation for many schools. This is where crucial role modelling occurs, so is an area where input is needed (MEL 6) More resourcing for 1–1 sessions would be ideal, as developing empathy can be deeply rooted in the individual's own experiences, anxieties, & insight. (MEL 6) To remain empathic you must feel valued and supported. This extends to post grad therefore system wide role modelling of empathy is needed not just with patients (MEL 24) More time and effort should be spent in ensuring our future doctors can be empathetic to their patients. (MEL 5) It would be great to translate it into the clinical environment however that would involve training all the clinicians who are involved in teaching medical students (MEL 25) This [training] takes time, reflection and input as they [students] mature and accumulate life experience. This is a process that packed curricula don't easily support. (MEL 6)
Student attitude, experience and engagement	Whilst things have improved for empathy/communication, there are still a reasonable proportion of students who perceive these areas as soft and fluffy, and not as important as the hard science. (MEL 5) Students with learning difficulties can have particular challenges relating to others, which may need specialist support (Asperger's being one example). This is important, but resource intensive (MEL 6)
Staff and faculty attitudes and experience	Development of empathy I think needs to be positioned as a life-long process, not something we "teach in this session" MEL 6 Empathy cannot be taught but is nurtured and grows over time. (MEL 11) The value (wanting to understand other perspectives) should drive the behaviour. We need to shift from an assessment led approach ('what to I need to say in the OSCE') to a values based approach from day 1 ('what sort of doctor do I want to be and what do I need to do to get there'). (MEL 6) Also we need to recognise that it is developing the value that is paramount (not students 'learning what to say/do' at surface level). This takes time, reflection and input as they mature and accumulate life experience. This is a process that packed curricula don't easily support. (MEL 6) The simulated patient sessions cross-years pick this up [empathy], but improvements are needed in consistency of tutor approach. SP sessions taking place in GP practices, e.g., rely on large numbers of clinical colleagues whose approaches/focus may differ (MEL 6) Personally, I wouldn't feel it would be appropriate to routinely use research measures of empathy in medical school teaching and assessment activities. (MEL 19) empathy is not disaggregated from other skills (and, I would argue, is very difficult to either teach or examine in isolation). (MEL 9) I do a lot of exploring empathy in my interviews but I'm not sure it happens in all colleges (MEL 9)

#### Empathy-focused training and development

MELs described how clinical empathy is taught through their curriculum. They describe varying levels of integration, from specific, bespoke interventions that sit separately from other activities, to activities with the primary aim of fostering empathy, to an assumption that empathy training is covered somewhere in the curriculum.

(i) Dedicated empathy-focused training and development.

MELs discussed standalone empathy training that aimed to be patient-centred, encouraging students to focus on ‘understanding the patients’ perspective’, identifying the ‘therapeutic nature’ of consultations and becoming more ‘attuned to their patients’.

*“The course “Developing Clinical Empathy” aims to help students develop an empathic practice that is personal and attuned to their patients.”* (MEL 1)

*“There is a 1 hour online workbook that outlines why we should be empathetic to our patients…”* (MEL 5)

*“Specific simulated patient encounters to deal with empathy*” (MEL 16)

(ii) Empathy training is integrated into other activities.

MELS stated that specific learning on empathy was integrated into other teaching activities, including communication skills, clinical placements and through specific scenario training such as in ‘breaking bad news’.

*“we discuss empathy and the use of empathetic statements routinely as part of our small group communication skills training.”* (MEL 20)

*“empathic communication is integrated into pretty much all of the communication training, which occurs throughout the course.”* (MEL 14)

(iii) Empathy training is intrinsically taught through other activities.

A number of MELS described how they felt empathy was nurtured in students indirectly, through a number of other teaching and learning activities, without the need to necessarily focus on empathy as a specific skill in itself.

*“I think it is very difficult to untangle whether the primary aim of a teaching session is to improve empathy – it is taught with other skills and where the emphasis lies depend on who is teaching.”* (MEL 9)

*“we don’t label anything specifically as ‘empathy training’ but we include the above [definition of empathy as used by survey] which contribute to this construct”* (MEL 7)

(iv) Assumption that empathy training is included elsewhere in the curriculum.

There was a belief from some MELs that empathy development in students was automatically incorporated into other curricular activities. Some were unclear however if this did indeed happen consistently or to what extent it occurred.

*“All clinical placements should have inbuilt opportunities to further develop clinical empathy, but I am unsure how much this occurs.”* (MEL 6)

*“We don’t specifically say “empathy-based training” as we are not teaching students to ‘learn to sound empathic’ as an isolated skill, or thinking about ‘one item’. It is - hopefully - integrated.”* (MEL 6)

*“I suspect this is provided on an ad hoc basis in a variety of specialties. It is informally being delivered throughout the curriculum.”* (MEL 17)

#### Challenges presented in putting empathy on the curriculum

MELs commented frequently on the challenges they found from their experience, or could foresee in attempting to further establish empathy-focused training on the undergraduate curriculum. Three sub-themes were identified here, with challenges related to the system, students or staff.

(i) System-based challenges.

Lack of time and resources, large cohorts of students, poor role models and lack of control over clinical environments were all cited as potential problems when trying to implement training for students in empathy.

*“It would be great to translate it into the clinical environment however that would involve training all the clinicians who are involved in teaching medical students.”* (MEL 25)

*“Scale is a challenge to standardisation for many schools. This is where crucial role modelling occurs, so is an area where input is needed.”* (MEL 6)

(ii) Student engagement, experience or attitude.

MELs commented on student engagement with and attitude to training and the difficulties this presents. In addition, the need for ‘specialist support’ for students who have ‘particular challenges relating to others’ was raised as a concern.

*“Whilst things have improved for empathy/communication, there are still a reasonable proportion of students who perceive these areas as soft and fluffy, and not as important as the hard science.”* (MEL 5)

(iii) Staff and faculty engagement, experience or attitude.

Attitudes to empathy-focused training and how best to implement it varied with some MELs rejecting the idea that empathy can be taught. Concerns were raised that training and assessment would drive students away from a ‘values based approach’ with students more concerned about appearing empathic to pass exams.

*“The value (wanting to understand other perspectives) should drive the behaviour. We need to shift from an assessment led approach (‘what do I need to say in the OSCE’) to a values based approach from day 1 (‘what sort of doctor do I want to be and what do I need to do to get there’).”* (MEL 6)

*“Empathy cannot be taught but is nurtured and grows over time.”* (MEL 11)

#### Survey of online materials

All 41 medical schools’ online materials provided information about undergraduate teaching and the course curriculum for their degree programme(s). Information obtained from websites/online prospectuses was summarised (see Additional file 3). The majority (83%) of medical schools described no specific empathy-focused teaching/learning activities explicitly aimed at enhancing empathy in their degree course. Seven medical schools described some form of teaching/learning that was either labelled as empathy-focused, aimed at fostering empathy or described as an activity that could be considered to fit with our definition of empathy [19]. For example, one medical school described a teaching activity as “giving our students invaluable insight into the experience of people with a medical condition or disability, and their carers”. There was variation in the descriptions given, with some online materials mentioning empathy as an outcome or aim of teaching, and others giving more specific detail about what/when/where empathy training is provided. We accept that some teaching/learning activities that could be considered to foster empathy may be described without explicitly discussing empathy. Twenty-six medical schools (63%) explicitly referenced empathy as part of the selection process to medical school, with the majority referring to empathy being assessed during Multiple Mini Interviews.

Of the 28 schools that responded to our survey, 23 (82%) websites described no specific empathy-focused teaching/learning activities. Eighteen (64%) explicitly referenced empathy as part of the selection process. A review of the websites of the 11 schools that did not respond to the survey identified that nine (82%) described no specific empathy-focused teaching/learning activities. Five (45%) explicitly referenced empathy as part of the selection process to medical school.

## Discussion

### Summary and evaluation of results

This study is the first of its kind to explore the current provision of empathy-focused training at UK medical schools through a survey of medical educators and of medical school websites. The results of this survey provide valuable insight into the priority that clinical empathy training is currently given at medical schools across the UK. Our main finding was that while most respondents report that their undergraduate curriculum includes empathy training, empathy is rarely assessed, and there is an appetite for more.

Our results highlight a number of discrepancies between different aspects of the quantitative results, and between the quantitative and qualitative results.

Firstly, whilst the majority of medical schools report they provide some form of empathy-focused training to students, most do not provide dedicated empathy-focused training, programmes or modules. Almost two thirds of medical schools believed that their curriculum provides empathy training labelled as something else (with examples including communication skills, consulting skills, medical humanities, mindfulness, ethics and professionalism). Given the general lack of consensus in defining empathy in the clinical setting, it is unsurprising that there should be confusion over what empathy-specific training is and what it is not. Our thematic analysis supports quantitative findings in identifying that there is some confusion or misunderstanding around if, when and where empathy-focused training is delivered in the curriculum. For example, some educators assume empathy training is integrated throughout the curriculum or intrinsically taught through other activities, though some noted it cannot be assumed that empathy training is implied in the curriculum. Our findings support those of a recent systematic review [27] which found that interventions aiming to improve medical students’ interpersonal communication skills, targeting skills associated with empathy but not specifically empathy-focused, do not always improve student empathy. In addition, evidence supports the need for empathy-focused training to be delivered as a formal, sustainable programme integrated into the curriculum [8, 28] if it is to be impactful.

Secondly, whilst almost all medical schools reported that they do include some form of empathy-focused training, a third of these did not report or were unsure if ILOs are associated with empathy-focused training. Relatedly, approximately a third reported either that there is no formal evaluation of empathy-focused training or uncertainty regarding whether there is formal assessment. Two-thirds of medical schools reported that there is either no form of training provided to faculty around how to teach clinical empathy or that they are unsure.

Thirdly, whilst the majority of medical schools (80%) reported that students’ ability to empathise is assessed (formally or informally, for example through OSCEs or portfolio activities), only one medical school reports the use of a validated empathy-specific measurement tool. Research identifies that in order to move empathy from a nebulous idea to a tangible skill, students must be evaluated and empathy assessed [29]. Whilst there is currently little research into the role of student assessment on the formation and development of empathy at medical school, evidence from the wider field of medical education suggests formal assessment and evaluation can support students in achieving the desired outcomes [30].

Lastly, most wish to see more training offered. This tentatively implies that they believe that current empathy training is lacking.

Our website search identified that with a small number of exceptions, most medical school websites do not describe empathy-focused training explicitly as part of the content of their undergraduate medical degree.

### Strengths and limitations

To the best of our knowledge, this is the first study to explore the current provision of empathy-focused training at UK Medical Schools. A strength of the study is our high response rate. There are also several limitations to this study. Our focus has been on UK medical schools, which limits the generalisability of our findings. Investigation as to whether empathy training is included in undergraduate curricula more internationally may provide useful insights into how different countries support their students in fostering an empathic approach to practice, and the importance they give it. Another limitation is that our study relies on the concept of empathy, which remains poorly defined. The thematic analysis identified that participants felt empathy-focused training is provided indirectly through other teaching and learning activities, such as communication skills. A final limitation is our website search. This was performed by one person and relied on search terms, and therefore we may have missed mentions of empathy. In addition, the website search limitations correlates to other limitations, especially that empathy could be described on the website but called something else (such as communication skills). By describing the findings of a the website search by schools who did and did not respond to our survey, we sought to identify whether a respondent bias was present. There was little difference between the websites of schools who responded and those who did not in terms of whether empathy-focused training is explicitly discussed on the school website (82% of schools who did not respond and 82% of schools who did respond to the survey did not explicitly describe empathy-focused training/learning activities). A minor difference from the websites of responders and non-responders was that more of the schools that responded referenced empathy as part of the selection process (64% versus 45%). We therefore cannot rule out that non-responding schools could put less focus on the role of empathy training in medical school. Of note, our high response rate reduces the potential impact of responder bias.

### Implications for research and practice

Medical educators have clearly expressed a strong appetite for more empathy training in medical schools. To achieve this, further research is needed to clarify the definition of empathy in the healthcare setting. This could then support the identification of the most appropriate pedagogical approaches or educational tools to nurture therapeutic empathy. In addition, whilst it is unlikely that a single ‘one-size-fits-all’ package of empathy training would be beneficial, evidence-based strategies will guide educators in making the most of often limited space on the curriculum.

## Conclusion

Our survey identifies that empathy-focused training is included to some extent on the medical school curricula of most UK medical schools. There is confusion around how therapeutic empathy and empathy-focused training differs from other related, but different concepts (such as compassion or communication skills). Empathy-focused learning outcomes are not always identified, training not consistently evaluated and student empathy not specifically assessed. The results from this survey supports evidence that there is an appetite for further investment in empathy-focused training at medical school, [31] but that there is little agreement at present on how best to proceed. A consensus on the definition of intended learning outcomes encompassing clinical empathy would facilitate a clearer comparison of empathy teaching and its evaluation and assessment in different courses.

## Supplementary information

**Additional file 1:** Survey questions.

**Additional file 2: **Participant invite and survey description.

**Additional file 3: **Summary of empathy-focused training provided by UK medical schools websites/online materials.

## Data Availability

The datasets used and analysed during the current study are available from the corresponding author on reasonable request.

## Abbreviations

MEL: Medical Education Lead
ILO: Intended Learning Outcomes
OSCE: Objective Structured Clinical Examination
