# Supplementary material for: A survey of clinical empathy training at UK medical schools
Source: BMC Med Educ. 2023 Jan 19;23:40. doi: 10.1186/s12909-022-03993-5 (PMC9850684; doi:10.1186/s12909-022-03993-5)
Supplement: Supplementary file 1 — Additional file 1: Survey questions. [file 12909_2022_3993_MOESM1_ESM.pdf]

A survey of clinical empathy training at UK medical schools

Page 1: Survey Information

We are undertaking a survey of ***clinical empathy training*** provided to medical students as part of the curricula at UK medical schools. We are extremely grateful to you for taking the time to complete this survey.

We realise that all communication skills training and some other types of training enhance empathy to some degree. For the purpose of this study, however, we would like to **focus on things that explicitly aim to enhance clinical empathy.** For this study, we take ***'empathy-focused training'*** to mean ***any educational activity with the primary aim of fostering skills in clinical empathy.***

We recognise that there are a variety of definitions of empathy, and you will have a chance to comment at the end of the survey. For the purposes of this study, we take ***clinical empathy*** to mean three things:

- the ability to understand the patient's situation, perspective and feelings (and their attached meaning)
- to communicate this understanding and check its accuracy, and…
- to act on it in a helpful (therapeutic) way.

We appreciate your awareness of the content of the entire curriculum at your medical school may be limited, but please answer questions to the best of your knowledge.

By undertaking this survey you are giving permission for the data you provide to be used for research purposes. Any data used for research purposes will be anonymised.

Page 2: Privacy note

Data collected from this survey will be transferred from the online portal to an excel spreadsheet. All data will be stored in a secure University Research Directory.

Page 3: Medical School and degree programme

1. Name of Medical School

2. What type of degree programme(s) does your medical school offer? *Please select all that apply (Course type options as described by the Medical Schools Council)*

- ☐ Standard Entry Medicine (undergraduate degree)
- ☐ Graduate Entry Medicine
- ☐ Medicine with a Gateway or Foundation Year
- ☐ Medicine with a Preliminary Year

3. Does your medical degree programme offer a foundation or gateway year?

- ○ Yes
- ○ No

4. Is your curriculum taught at another medical school?

- ○ Yes
- ○ No

4.a. If Yes, please specify which

4.b. Which of the following best describes what you do?

- Curriculum design
- Curriculum delivery (teaching)
- Curriculum design and delivery
- Other

4.b.i. If other, please specify

Page 4: Empathy-focused training

5. Please state whether you are answering these questions based on an undergraduate degree curriculum, a graduate entry degree curriculum or both?

- Standard (undergraduate) degree curriculum
- Graduate entry degree curriculum
- Both

6. Does your curriculum include formal empathy-focused training (educational activities that have been specifically designed to foster clinical empathy)?

- Yes
- No
- Unsure

6.a. If yes, is there a dedicated empathy-focused training programme/module in your curriculum, or are empathy-focused training activities integrated across other courses or modules?

- There is a dedicated empathy-focused programme or module
- Empathy-focused training activities are integrated into other courses or modules
- Both of the above
- Unsure

6.b. If your curriculum does include empathy-focused training, have specific empathy-focused intended learning outcomes been developed associated with this?

- Yes
- No
- Unsure

7. If your curriculum includes specific empathy-focused training, when does it take place? *Please select all that apply*

- [ ] Foundation/gateway year
- [ ] Year 1
- [ ] Year 2
- [ ] Year 3
- [ ] Year 4
- [ ] Year 5
- [ ] Other

7.a. If you have answered 'other', or none of the options are suitable, please describe when/where empathy-focused training is offered

8. If specific empathy-focused training activities are provided, are they part of the compulsory curriculum for all students or offered on an elective basis (for example, as part of a student-selected component)?

- ○ Compulsory
- ○ Elective
- ○ Both
- ○ Unsure

9. If empathy-focused training is provided, what teaching methods are employed to deliver this? *Please select all that apply*

- ☐ Lectures
- ☐ Problem-based learning
- ☐ Seminars
- ☐ Small group work
- ☐ Online activities
- ☐ Clinical experiences
- ☐ Other

9.a. If other, please describe

10. If empathy-focused training is provided, who is responsible for the delivery of it? *Please select all that apply*

- ☐ Clinical academics
- ☐ Academics
- ☐ NHS clinicians
- ☐ Clinical teaching fellows
- ☐ Patients
- ☐ Other

10.a. If other, please describe

11. Does your medical school provide any form of training or development for faculty and clinical educators around clinical empathy and teaching this to students?

- Yes
- No
- Unsure

11.a. If yes, please describe what

12. Please describe in your own words what empathy-focused training (educational activities with the primary aim of fostering empathy in students) your medical degree programme provides

13. Is there anything that fits our definition of clinical empathy (the ability to understand the patient's situation, perspective and feelings, communicate this to them, and act on it in a helpful and therapeutic way) which you feel your curriculum delivers, but is labelled as something other than empathy-focused training?

- Yes
- No
- Unsure

13.a. If yes, please describe

Page 5: Assessment of student empathy

14. If empathy-focused training is offered, is it evaluated?

- Yes
- No
- Unsure
- Empathy-focused training is not offered

15. If empathy-focused training is offered, is student feedback sought?

- Yes
- No
- Unsure
- Empathy-focused training is not offered

16. Is student empathy assessed at any point during the degree programme?

- Yes
- No
- Unsure

16.a. If yes, how is it assessed? *Please select all that apply*

- Self-assessment
- Reflective practice
- Written exam
- OSCE
- Portfolio activities

- [ ] Other

16.a.i. If other, please describe

16.b. Are any empathy-specific tools used to measure student empathy? *Please select all that apply*

- [ ] None
- [ ] Barrett-Lennard Relationship Inventory-empathy understanding (BLRI)
- [ ] Consultation and Relational Empathy (CARE) measure
- [ ] Empathy Construct Rating Scale (ECRS)
- [ ] Empathy Quotient (EQ)
- [ ] Jefferson Scale of Empathy (JSE)
- [ ] Medical Condition Regard Scale (MCRS)
- [ ] Reynolds Empathy Scale (RES)
- [ ] Therapist Empathy Scale (TES)
- [ ] Toronto Empathy Questionnaire (TEQ)
- [ ] Other

16.b.i. If other, please specify

17. Is the assessment of empathy, or a student's ability to empathise considered in the admissions to medical school process (for example at the interview stage)?

- [ ] Yes
- [ ] No
- [ ] Unsure/unable to say

17.a. If yes, please describe how/when

Page 6: General

18. Do you think clinical empathy should be taught at medical school?

- Yes
- No

18.a. If yes, do you have any suggestions for how clinical empathy should be taught (or, if it is taught at your institution, how teaching could be improved)?

19. Do you think your medical school does enough to foster clinical empathy in medical students?

- Yes
- No
- Unsure

20. Would you like to see more empathy-focused training on your undergraduate curriculum?

- Yes
- No

21. Do you have any other comments you think are relevant?

22. If you would be willing to be contacted to discuss this survey and empathy-training at medical school further, please leave your contact details below.

Page 7: Final page

Thank you very much for completing this survey.

We intend to use the results of this survey to understand to what extent empathy training is currently imbedded in UK medical school curricula, and whether medical educators believe this is a training need still to be met.
