# Supplementary material for: A survey of clinical empathy training at UK medical schools
Source: BMC Med Educ. 2023 Jan 19;23:40. doi: 10.1186/s12909-022-03993-5 (PMC9850684; doi:10.1186/s12909-022-03993-5)
Supplement: Supplementary file 2 — Additional file 2: Participant invite and survey description. [file 12909_2022_3993_MOESM2_ESM.docx]

**Additional File 2. Participant invite and survey information**

**Email sent to Medical Education Leads**

Dear xxxxx

**Re: Survey of empathy-training in undergraduate medical education**

As part of my PhD, and working with colleagues from Leicester Medical School, I am undertaking a survey of clinical empathy training provided to medical students as part of the medical curricula at UK medical schools.

There is evidence that practitioners who enhance the way they express empathy improve patient outcomes ([for example by improving pain a little](https://journals.sagepub.com/doi/full/10.1177/0141076818769477)) and can [reduce their own burnout](https://www.ncbi.nlm.nih.gov/pmc/articles/PMC3631218/). Research shows that [training in clinical empathy is effective.](https://bmjopen.bmj.com/content/bmjopen/10/9/e036471.full.pdf)

We realise that all communication skills training and some other types of training enhance empathy to some degree. However, for the purpose of this study we would like to focus on things that explicitly aim to enhance clinical empathy. For this study, we take ‘empathy-focused training’ to mean any educational activity with the primary aim of fostering skills in clinical empathy. We take [clinical empathy](https://www.ncbi.nlm.nih.gov/pmc/articles/PMC1316134/) to mean three things:

- the ability to understand the patient’s situation, perspective and feelings (and their attached meaning)
- to communicate this understanding and check its accuracy, and…
- to act on it in a helpful (therapeutic) way.

This online survey should take no longer than 15 minutes to complete. We appreciate your awareness of the content of the entire curriculum at your medical school may be limited, but please answer questions to the best of your knowledge.

We intend to use the results of this survey to understand to what extent empathy training is currently imbedded in UK medical school curricula and whether medical educators believe this is a training need still to be met.

By undertaking this survey, you are giving permission for the data you provide to be used for research purposes. Any data used for research purposes will be anonymised.

If you would like more information or would like to discuss anything further please do contact me.

Yours sincerely

**Dr Rachel Winter**

*Academic Clinical Lecturer in medical education and old age psychiatry*

Leicester Medical School

College of Life Sciences

University of Leicester | University Road | Leicester | LE1 7RH | UK

**t:**  xxxxxxx

**e:**  xxxxxxx
**w:** xxxxxxx

Participant information

**Re: Survey of empathy-training in undergraduate medical education**

As part of my PhD in medical education, I am working with Dr Andy Ward at Leicester Medical School to undertake a survey of clinical empathy training provided to UK medical students as part of the undergraduate medical curricula.

The survey should take no longer than 15 minutes to complete. We appreciate your awareness of the content of the entire curriculum at your medical school may be limited, but please answer questions to the best of your knowledge. Alternatively, if you know someone who would be better placed to complete the survey, we’d very much appreciate you passing the request on.

Link to the survey:

https://leicester.onlinesurveys.ac.uk/a-survey-of-clinical-empathy-training-at-uk-medical-school-3

There is evidence that practitioners who enhance the way they express empathy improve patient outcomes ([for example by improving pain a little](https://journals.sagepub.com/doi/full/10.1177/0141076818769477)) and can [reduce their own burnout](https://www.ncbi.nlm.nih.gov/pmc/articles/PMC3631218/). Research shows that [training in clinical empathy is effective.](https://bmjopen.bmj.com/content/bmjopen/10/9/e036471.full.pdf)

We realise that all communication skills training and some other types of training enhance empathy to some degree, however, for the purpose of this study we would like to focus on things that explicitly aim to enhance clinical empathy. For this study, we take ‘empathy-focused training’ to mean any educational activity with the primary aim of fostering skills in clinical empathy. We take [clinical empathy](https://www.ncbi.nlm.nih.gov/pmc/articles/PMC1316134/) to mean three things:

- the ability to understand the patient’s situation, perspective and feelings (and their attached meaning)
- to communicate this understanding to them and check its accuracy
- to act on it with them, in a helpful (therapeutic) way

We intend to use the results of this survey to understand to what extent empathy training is currently imbedded in UK medical school curricula and whether medical educators believe this is a training need still to be met. By undertaking this survey, you are giving permission for the data you provide to be used for research purposes. Any data used for research purposes will be anonymised.

If you would like more information or would like to discuss anything further, please do contact us.
