# Supplementary material for: A survey of clinical empathy training at UK medical schools
Source: BMC Med Educ. 2023 Jan 19;23:40. doi: 10.1186/s12909-022-03993-5 (PMC9850684; doi:10.1186/s12909-022-03993-5)
Supplement: Supplementary file 3 — Additional file 3: Summary of empathy-focused training provided by UK medical schools websites/online materials. [file 12909_2022_3993_MOESM3_ESM.docx]

**Addition file 3: Survey of medical school websites**

Search strategy*: Manual search of the University website, medical school website/webpages, online/downloadable prospectuses (if available) and programme specification (if available) for any information related to empathy-focused training (or training related to compassion/compassionate care) and general communication and interpersonal skills training/learning. Search of University website using terms ‘empathy’ ‘empathic’ ‘empathetic’ ‘compassion’ and ‘compassionate’

| **Name of medical School and course type** offered** | **Information related to interpersonal and communication skills training as supplied by website/course pages/prospectus/programme specification (where available)** | **Information identified relating specifically to empathy-focused teaching/learning outcomes** | **Admissions and selection information related to empathy** | **Information related to empathy and empathy-related terms using search function on university website** |
| --- | --- | --- | --- | --- |
| **University of Aberdeen School of Medicine and Dentistry**  SEM  MFY/GY | “Early patient contact…work with our Patient Partners right from the very start…”  Year 1: “introduction to the practical and communications skills that will form the foundations of your medical practice.”  Year 3: “The Medical Humanities SSC [student selected component] in year 3 gives you an opportunity to acquire an alternative ‘take’ upon medicine and/or health, sickness or disability, which is not provided by the mainstream medical curriculum” | Medical Humanities SSC with Creative Writing for Medical Students option to allow students to “use the creation of fiction and poetry as a means to heighten perception, empathy, and awareness of the experience of themselves and others within a medical environment” “We feel this course enriches students' personal lives as well as their educational experience, allowing for a greater understanding of patients' lives.” | Application process, ‘things to consider’ in writing personal statement includes: “Empathy and the ability to care for others.” |  |
| **Anglia Ruskin University School of Medicine**  SEM | Phase 3: “‘Learn to look after the patient as a whole’ focus on ‘holistic care’”  “Patient Partners…volunteers who provide invaluable role in the development and training of our students...so that students can develop both their communication skills…” | No specific information relating to empathy-focused teaching/learning provided or learning outcomes focused on empathy | Selection process - Interview includes tasks assessing ‘interpersonal and communication skills (including empathy)’ |  |
| **Aston University Medical School**  SEM | A ‘patient focused’ curriculum.  “students [are] encouraged to think always about what it is like for the patient, to be empathic to their situations and concerns…”  There is “…an emphasis on working compassionately with patients as partners..”  Phase 1: students learn “Communication skills, interacting with patients/professionals” | No specific information relating to empathy-focused teaching/learning provided or learning outcomes focused on empathy | Through selection process “We’re looking for qualities such as… Empathy, compassion, respect and dignity” |  |
| **Barts and The London School of Medicine and Dentistry**  SEM  GEM | There is “considerable emphasis on developing your expertise in a whole range of practical areas, including clinical, communication, observation, team-work and management skills.”  “One of the most important skills you’ll need to develop is teamwork and the ability to communicate well with colleagues, patients and relatives.”  “emphasis on communication skills throughout the programme”  “As a student, you’re exposed to clinical situations from very early on, and where learning communication skills is a primary focus of teaching.”  “We also have a purpose-built Clinical and Communications Skills Centre…The Centre is one of the most advanced of its kind and the first to be developed in the UK, which means you can develop the key communication skills you’ll need with the help of specially trained actors who simulate patients and are able to give valuable feedback.”  Phase 3: “individual sessions in communication skills teaching and simulated patient scenarios.” | No specific information relating to empathy-focused training/learning or learning outcomes focused on empathy | Empathy not specifically mentioned as skill/attribute in selection process |  |
| **University of Birmingham College of Medical and Dental Sciences**  SEM  GEM | “We teach you the practical, theoretical and communication skills needed to become a safe, compassionate and ethical doctor of the future.”  “you will graduate as a smart, practical, compassionate, safe and ethical doctor, renowned for your professionalism and communication skills and excelling at making a difference to other people's lives.”  Student will “see a vast range of illness in people from diverse ethnic backgrounds, genders, sexualities, socio-economic statuses, and ages, helping you to relate, empathize and identify with these people on a professional and personal level.”  The course will develop “Expert communicators who are empathic in a manner appropriate to a caring profession” | No specific information relating to empathy-focused training/learning or learning outcomes focused on empathy | Selection process “MMIs [multiple mini interview] allow you to demonstrate a range of skills you have which are relevant to studying medicine, such as…empathy” |  |
| **Brighton and Sussex Medical School**  SEM | “By the time you graduate, you will have developed the knowledge and key personal skills and attitudes necessary to pursue a successful medical career.”  “Patient communication and clinical skills are integral to your studies at BSMS [Brighton and Sussex Medical School]. Working with professional actors, our simulation rooms will allow you to practise patient consultations, preparing you to manage complex problems”  Year 1 &2: “You will start to develop clinical skills in…effective communication with patients in a classroom setting and by gaining experience in primary, secondary and third sector placements. For example, you may spend time with a family looking after a new baby (Year 1), and with a patient with dementia or a chronic health condition (Years 2 and 3).”  “You will spend time with a person with dementia and their carer, getting to know them and seeing how the disease affects them and their family.”(*Time for dementia project*)  “Clinical symposium sessions with scientists, clinicians and patients exploring a medical condition from different perspectives”  Year 3: Student will have “Regular meetings with your tutor will support your personal and professional development”  Year 4: Students will participate in the *Time for Autism* project | *Time for Dementia* “conceived to provide undergraduate healthcare students with understanding, empathy, knowledge and compassion to support families living with dementia in the future.”  *Time for Autism* programme “Future doctors who come into contact with autistic people and their families need to be equipped with the appropriate skills, knowledge, empathy and understanding to provide good quality care, regardless of their clinical specialism.” | Film of example MMI station “tell me, what do you understand by the term empathy” |  |
| **University of Bristol Medical School**  SEM  MFY/GY | Year 1: “provide opportunities for students to meet with patients and discuss their health and well-being”  “initiate training in medical communication skills and use of medical terminology”  Year 2: “continue the development of communication skills and effective consultation.” “Allow students to meet patients and discuss their disease and how it impacts on them”  Year 4: “facilitate a holistic understanding of the experiences and needs of patients and their carers.” “Encourage students to be self-reflective and further develop their professional identity and behaviour”  Year 5: “To allow students to meet patients and their carers (where appropriate) to discuss their disease and how it impacts on them in both hospital and community settings”  MGY: “Encourage self-awareness and reflective thinking, leading to the identification of strategies for continued development.”  “Demonstrate familiarity and confidence with the development and application of core skills including communication, team-work and time management.”  “Reflect on and assess the attributes and professional behaviours necessary for a successful career” | “CMOP (Complex Medicine in Older People) and PCC (Primary Care and Community) placements are longitudinal clerkships which will provide continuity of place of learning, tutor and patients. This enables students to participate in patient care over time, helps students to maintain empathy with patients, promotes clinical learning and enhances professional development.  Learning outcomes from programme specification include:  “Respect patient autonomy and adopt a holistic and empathetic approach to the patient/doctor relationship”  “Bristol graduates will possess all the core competences, knowledge and practical skills to enable them to enter the workplace as safe, effective and compassionate practitioners.” | Empathy not specifically mentioned as skill/attribute in selection process |  |
| **University of Buckingham Medical School**  SEM (4.5 years) | “…produce highly ethical, honest and compassionate doctors who will put the patient first.”  Phase 1: “enable students to carry out a patient-centred consultation…and understand the importance of the patient perspective in diagnosing and managing patient problems”  Narrative Medicine unit “enable students to gain an insight into the patient's perspective” “developing the ability to reflect on experiences as a basis for life-long learning.”  “our students will be able to communicate effectively with patients”  SSC Medical Humanities (optional): “the importance of visual and/or verbal storytelling in communicating health narratives and inculcating compassion” | Narrative medicine course with aim of enabling “students to gain an insight into the patient's perspective”  No other specific empathy-focused learning outcomes identified | Empathy not specifically mentioned as skill/attribute in selection process |  |
| **University of Cambridge School of Medicine**  SEM  GEM | “our aim is to educate students to become compassionate, thoughtful, skilled members – and leaders – of the medical profession.”  “We enable students to develop the excellent communication, clinical, interpersonal and professional skills required for good medical practice.”  Year 1-3:Preparing for Patients module “prepare them for clinical study by starting to develop the communication skills they will need to interact successfully with patients.” | No specific information relating to empathy-focused training/learning or learning outcomes focused on empathy | “Key criteria for medical admissions… Listening and empathy skills and an ability to interact with others, both professional and lay.” |  |
| **Cardiff University School of Medicine**  SEM  GEM | “Our aim is to produce great clinicians who understand people and the environment in which we live”  Phase 1 (Year 1 and 2): ““develop the fundamental communication, clinical skills and professionalism required of a doctor.”  Phase 2 (Year 3 and 4): “expert teaching on…clinical and communication skills.” “provide you with an insight into the patient experience of both illnesses and the health care system.” “The patient should remain the focus of the learning”  Phase 3 (Year 5): ‘Harmonisation Programme’ assistantship. “Core learning on campus covers advanced aspects of professionalism, communication…so that you are ready to work as an F1 doctor.” | No specific information relating to empathy-focused training/learning or learning outcomes focused on empathy | Empathy not specifically mentioned as skill/attribute in selection process |  |
| **University of Dundee School of Medicine**  SEM  MFY/GY | Simulation-based learning to “build your knowledge, confidence, people skills, and resilience”  Course aims to produce “confident, able doctor, prepared for practice, by developing a flexible, patient-centred style of care, underpinned by scientific and clinical knowledge” | No specific information relating to empathy-focused training/learning or learning outcomes focused on empathy | Empathy not specifically mentioned as skill/attribute in selection process |  |
| **Edge Hill University Medical School**  SEM  MFY/GY | Non-modular curriculum approach with 16 themes including communication and interpersonal skills “how to communicate openly, honestly and effectively with patients and their relatives, carers and other advocates, as well as with colleagues.”. Focus on “understanding the person and the community” and patient-centred care.  MFY: Core themes including professional and personal development and communication “different styles and methods of communication; barriers to effective communication; communication skills with patients, colleagues and carers; written and academic communication for practice; models and theories of communication; patient-centred communication” | No specific information relating to empathy-focused training/learning or learning outcomes focused on empathy | MMI designed to assess empathy and compassion, amongst other things. |  |
| **The University of Edinburgh Medical School**  SEM (6 years) | Year 1: ‘Whole body health’ module ‘Clinical and communication skills sessions and problem-based learning will also be used to explore more deeply both knowledge and approach to learning.” “You will gain an understanding of: communication, empathy, compassion and wellbeing.”  “You will have patient contact from semester 1, focusing on patients’ experience of illness and the influence of social factors”  Year 2: ‘The importance of communication, empathy, compassion and wellbeing are covered under Professional Skills and will be evidenced in your online Clinical Portfolio’  Year 3: Option to study medical humanities as part of the honours programme to obtain BSc | ‘Clinical and communication skills sessions…You will gain an understanding of: communication, empathy, compassion and wellbeing.” | Students advised to consider factors when writing personal statement (not assessed) including: “Personal qualities and skills; examples may include empathy, interpersonal relationships and the ability to communicate.” | “in 2018/9, we designed and ran workshops for medical and nursing students on empathy, communication and self-care” (research abstract using VR for empathy training) |
| **University of Exeter Medical School**  SEM  MFY/GY | The curriculum provides a clinical focus that is patient-centred  Year 1: ‘you will learn about person-centred and patient-centred communication, core clinical skills such as history-taking and examination as well as professional and ethical behaviours.’  Year 2: ‘Your Professional Development groups will provide a supportive environment in which you can learn from your placement experiences and explore what it means to be a medical professional.’  Year 3: ‘continue to acquire the core clinical skills and professional behaviours that underpin person and patient centred clinical practice.’ | Learning and teaching: “On graduation you will be able to approach clinical problems holistically, have excellent communication skills, be empathetic and a good listener.”  Optional SSU (student selected units) include:  Opportunities to “consider how the study of the humanities offers insights into the lived experience of people experiencing lives very different from our own”  “A student project to explore how arts and activities can enrich the lives of people who are at risk of being socially isolated… building on their empathy via this project” (offered in 2017, unclear if still offered) | Medical student interview “which is designed to determine whether applicants have the non-academic qualities required to become a successful doctor. Examples include good communication skills, evidence of empathy and of reflectiveness.” |  |
| **University of Glasgow Medical School**  SEM | Phase 2 :(year 1 and 2) specifically mentions communication skills, clinical and professional skills. Early patient contact “communication skills start in year 1 and continue throughout the programme” | No specific information relating to empathy-focused training/learning or learning outcomes focused on empathy | Empathy not specifically mentioned as skill/attribute in selection process | Article – ‘incorporating empathy in training standards for healthcare professionals’ (undated) |
| **Hull York Medical School**  SEM  MFY/GY | “Becoming an Exceptional Clinician…designed a curriculum that focuses on the effective clinical and communication skills you will need to become an empathetic and confident practitioner right from the start.”  “In your communications skills sessions you will interview simulated patients… observe students in your group undertake consultations in real-time via a video-link – learning how to provide detailed, descriptive and non-judgmental feedback…state-of-the-art recording facilities allow you to capture and analyse your consultation, which you can use to help improve the quality of your skills”  “Our curriculum has been designed to ensure you develop a wide range of skills that you will use in patient consultations throughout your career, from physical and mental state examination, to history taking, clinical reasoning and communication.”  “During the first two years of the five-year Medicine programme, you will attend clinical and communication skills sessions twice per week.” “Peer physical examinations are non-invasive, and students find they are an effective way to learn and develop clinical and communication skills. Having your peers practise on you also allows you to understand and empathise with the patient’s perspective more deeply”  “In simulated patient interactions, you will observe as your peers undertake consultations with highly trained actors in real-time, providing detailed, descriptive and non-judgemental feedback, so you can learn from each other and perfect your consultation skills. Our state-of-the-art recording facilities allow you to capture and analyse your consultations.”  MGY: “you begin to develop the excellent communication skills and confident, empathetic approach to delivering care that are the hallmark of our graduates.”  “Longitudinal Integrated Clerkship… allows you to follow patients longitudinally over time…This continuity in patient interaction allows you to become truly involved in the patient journey and build long-term rapport, to the benefit of both yourself and patients alike.”  Hull York Medical School ‘a different kind of medical school’ short film: “our students are really skilled communicators.”  “The graduates of the programme care about patients. They understand the problems that people face and they’re empathetic, they can understand it from the patient’s perspective as well as from the doctors’ so I think our programme sets graduates up to have skills at a level above other medical schools.”  “We focus on this whole area of resilience…we’re thinking constantly about how to help them develop and get over the various barriers they may face.”  “We pride ourselves on producing confident, compassionate and work-ready doctors, whose training, grounded in patient-centred care, has armed them with the medical knowledge, empathy and resilience they need not only to thrive as doctors but to make a positive difference to the lives of their patients.” | No specific information relating to empathy-focused training/learning or learning outcomes focused on empathy | MMI interview process includes assessing for “personal qualities such as empathy, tolerance of ambiguity and resilience” |  |
| **Imperial College London** Faculty of Medicine  SEM | “The emphasis of our new MBBS programme is on the development of Professional Values and Behaviours, Professional skills, and Professional Knowledge…”  Phase 1 Lifestyle medicine. Options to take humanities and social science modules  “The curriculum of this course reflects the values of the NHS Constitution: “Working together for patients, respect and dignity, everyone counts, commitment to quality of care, compassion and improving lives” | No specific information relating to empathy-focused training/learning or learning outcomes focused on empathy | MMI process – criteria considered includes “empathy and resilience” |  |
| **Keele University School of Medicine**  SEM  MFY/GY | Spiral curriculum Includes “integrated communication and clinical skills” “academic and professional development” module  Year 1: “…combines a range of learning strategies, including early clinical experience, integrated communication and clinical skills teaching…”  Opportunities to intercalate in medical humanities.  Programme specification reports GMC graduate outcomes around communication interpersonal skills. | No specific information relating to empathy-focused training/learning or learning outcomes focused on empathy | MMIs assess for ‘empathy and insight’ | 2021 Teaching Innovation Project  “Use of the arts to develop empathy and support patient-centred care in pharmacy students” Unclear if now incorporated into standard curriculum |
| **Kent and Medway Medical School**  SEM | “The patient-centred focus of your programme will help you gain a broad understanding of patients’ experiences of a range of health conditions.”  “Through the curriculum you will learn how to…work in partnership with patients, relatives and carers settings for shared decision making:  Year 1 – person-centred practice: “Aims to enable students to appreciate the needs of their patients…” | No specific information relating to empathy-focused training/learning or learning outcomes focused on empathy | Empathy not specifically mentioned as skill/attribute in selection process |  |
| **King’s College London School of Medical Education**  SEM  MFY/GY  GEM | Theme of curriculum to develop ‘patient centred doctors.  Early clinical experience and communication skills in stage 1 (year 1) to patients with lived experience or simulated patients.  Stage 2: “You will also follow patients for prolonged periods of time to learn how to deliver whole-person care.” Placement in General Practice  GEM: “Inter-professional Education and team-based learning are an integral part of the course, developing your teamwork, communication, and an awareness of your ethical and professional responsibilities” | No specific information relating to empathy-focused training/learning or learning outcomes focused on empathy | Empathy not specifically mentioned as skill/attribute in selection process | Empathy and resilience in medical students “Over an eight-week pilot programme, medical students were taught the fundamental principles of good visual composition and abstract photography…they were invited to produce an artwork to represent their personal response to a patient consultation.” (article identified on search for empathy on university search engine)  Kings student wins “Shepley Parkin Empathy [Student] Award… empathy plays a key part in establishing the confidence of patients and increases the chances of effective healing.” 2021 |
| **Lancaster University Medical School**  SEM | “Doctors need to be able to communicate effectively with patients and their families in difficult times, to be their advocate and help inform their choices. In year one, you will study the evidence base around effective communication and start to develop your communication skills in a safe environment, through interaction with simulated patients (actors). From year two onwards, you will develop your communication skills further through interaction with real patients in hospitals and GP practices.”  Year 2: “Communication skills sessions take place throughout Year 2, which build on your learning from year one, complementing your increasing clinical experiences on placement.”  “through weekly training sessions in the Clinical Skills Centre, and you will undertake extensive communication skills training to prepare you for patient contact in years two to five.”  Year 3: “You will learn about common conditions, and their underlying pathology; you will learn the specific history-taking, examination and communication skills you need in the rotation’s speciality;” | No specific information relating to empathy-focused training/learning or learning outcomes focused on empathy | “Successful applicants to Lancaster Medical School come from a wide range of backgrounds but all have the motivation and academic ability to thrive at medical school and demonstrate empathy and respect for patients.” |  |
| **University of Leeds School of Medicine**  SEM  MFY/GY | MGY: “training in the medical sciences with a strong emphasis on experiential learning and communication with tutors, healthcare professionals, patients and service users.”  Year 1: “Your communication skills, with both patients and fellow professionals, will be developed through teaching and through clinical placements with multi-disciplinary teams.”  “challenging curriculum combines thorough training in the medical sciences with a strong emphasis on communication and practical skills.”  “Our Patient Carer Community contributes to teaching, learning and assessment, giving our students invaluable insight into the experience of people with a medical condition or disability, and their carers.” | No specific information relating to empathy-focused training/learning or learning outcomes focused on empathy | Empathy not specifically mentioned as skill/attribute in selection process |  |
| **University of Leicester Medical School**  SEM  MFY/GY | Phase 1 (year 1 and 2): Integrated, interdisciplinary modules related to human structure and function in health and disease. Early patient contact, community attachments, social and psychological context of medicine, communication skills Compassionate Holistic Diagnostic detective course, Healthcare Assistant Training  “The purpose of the compassionate, holistic, diagnostic, detective [CHDD] course is to help students learn how to interact effectively with patients, colleagues and other health professionals so that the care of patients may be optimised. This course aspires to bring students an understanding that effective interactions in the best interest of their patients include interactions between, not just patients, but other health professionals and where appropriate relatives… involves gathering altogether non-verbal and verbal communication in order to set the patient interaction in its social, cultural context.”  Communication skills: “To enable the student to develop their communication and interpersonal skills and give them the opportunity to understand the importance of these for consultations between doctors and patients, using simulation.”  Phase 2 (years 3 to 5): Apprentice style clinical placements in medicine, surgery and general practice, SSCs, specialty attachments, and extended foundation assistantships to prepare for foundation year posts  ‘Doctors bag’ resources: “At Leicester we focus on developing empathic and compassionate doctors”.  MFY: Taught alongside biological sciences students and includes elements specific to medicine. Patient contact and clinical skills training. Empathy-focused curriculum.  “Alongside the scientific knowledge, you will learn professional communication and examination skills. You will initially work with simulated patients – actors and volunteers – before developing your skills with real patients.” | **MFY:** A pioneering and unique aspect of the Medicine with Foundation Year its bespoke empathy-focused curriculum. Our innovative course fosters your clinical empathy right from the very start of your training. You will learn through a person-centred approach to medicine with the understanding that empathy is a core value that will be central to your future practice. This is important because we know that treating people with empathy improves their experience of healthcare and is beneficial to practitioners too.  Empathy training:  “You will start learning about medicine and patients at the start of the course. The Clinical Empathy Programme runs alongside the other modules and consists of a series of tutorials, lectures and seminars focused on understanding the role of clinical empathy in medicine. It also includes practical sessions on developing your communication skills.”  “The Medicine: The Patient module builds on the Clinical Empathy Programme and has a patient-centred approach. You will have the opportunity to spend time on hospital wards and in a GP surgery to expand your knowledge and practise the skills you are learning. You will be allocated a patient in the community to visit on a regular basis and will use your communication and empathy skills, and the medical knowledge you are acquiring, to better understand how patients experience illness and disease.” | MMI assessing (amongst other things) “Empathy and the ability to care for others” |  |
| **University of Liverpool School of Medicine**  SEM  MFY/GY | ‘Our graduates will be…able to apply a compassionate, evidence-based and patient-centred approach to their clinical practice.’  Year 1: ‘Communication for clinical practice sessions in small groups with simulated patients prepare students for the clinical placements.’  Year 1-5: Programme specification sets out communication skills teaching and assessment for each year of the degree  Knowledge and understanding: “the nature of good communication, the nature of health beliefs (including about alternative and complementary therapies), and how these affect people’s responses to health problems”  “Effective communication with colleagues, with patients and their families and friends, and with other people in the population about health issues is essential to competent medical practice, and is recognised as such by the GMC. Students undertake an integrated communication skills programme, designed to increase awareness of the skills already possessed through interacting with others.” | No specific information relating to empathy-focused training/learning or learning outcomes focused on empathy | Empathy not specifically mentioned as skill/attribute in selection process |  |
| **University of Manchester Medical School**  SEM  MFY/GY | “The course uses mixed learning methods, but the key Manchester approach is the study of themed case discussions through facilitated group activities to emphasise enquiry, discussion, self-education, and the development of critical faculties and communication skills - all essential skills for doctors.”  “You'll attend sessions in the Consultation Skills Learning Centre (CSLC), where you can consult with 'simulated patients' played by actors and practice clinical skills on colleagues and our SimMan.” | No specific information relating to empathy-focused training/learning or learning outcomes focused on empathy | Non-academic Information Form to be completed by applicants states “Doctors must be able to communicate and empathise with their patients. This is enhanced by some shared life experiences.” |  |
| **Newcastle University School of Medical Education**  SEM  GEM | “medical programme will prepare you for a career as a compassionate and skilled practitioner”  Early years: Case-led learning “will include clinicals skills tuition, hospital and GP visits, anatomy practicals, small group seminars, communication skill seminars and lectures.  Year 1: “Clinical and communication skills are the very foundation of clinical practice and you build on your existing skills in Year 1.”  “Without excellent communication skills, clinicians would struggle to take a history from their patients, determine the diagnosis and formulate (and communicate) a management plan. Newcastle graduates frequently refer to their excellent communication skills in supporting their preparation for practice.  Trust between the patient and doctor is one of the main cornerstones of being a successful practitioner and this requires you to be a skilful communicator.” | No specific information relating to empathy-focused training/learning or learning outcomes focused on empathy | MMI’s assess for “Empathy and self-awareness” |  |
| **Norwich Medical School**  SEM  MFY/GY | MFY: “students will learn how to work effectively within a team and develop communication skills.”  “We place great emphasis on coaching and developing your communication skills, relating to patients and their loved ones in a compassionate and understanding way.”  “During your training with us you will develop your communication skills incrementally starting with the essential building blocks such as, active listening and how to take a medical history, advancing through the course to master much more complex clinical communication tasks such as motivational interviewing, breaking bad news, discussing resuscitation orders and managing strong emotions such as anger and distress. You will be taught consultation skills using a blend of lectures and small group experiential sessions where experienced actors portray a wide variety of roles and simulate patients. We know from the feedback that we receive from former students that communication skills teaching helps equip you as a medical student for the early and continuing clinical practice that is integral to our particular course. We look forward to meeting and working with you in the future.”  “Developing the art of communication through our excellent consultation skills programme, supported by dedicated tutors and role-players”  “You will explore how teams can work in different scenarios where the focus is on cognitive impairment & communication difficulties across the lifespan. You’ll begin to reflect on your personal and professional development, and begin to explore how you will collaborate & work with other professions to provide integrated person-centred care. In the 2 hour session you may also have the opportunity to become a Dementia Friend.” | No specific information relating to empathy-focused training/learning or learning outcomes focused on empathy | Interviews:  “..we look for applicants to demonstrate at interview…an empathic and caring approach’. | 2021 article found on internal search engine: All medical students given virtual reality headsets ““Through the use of VR, we are also able to give students the unique opportunity to step into the shoes of their patients to experience the world from the patient’s perspective. This has the incredible potential to harbour empathy and improve students’ understanding of the challenges that accompany medical conditions like dementia.” |
| **University of Nottingham Medical School**  SEM  GEM  MFY/GY | Year 1 and 2: Integrated medicine module including ‘patient communication’  GEM: Personal and Professional development module “instruction and practice in the areas of basic communication & clinical skills and professional development as a doctor”  MFY: “introduction to the areas of basic communication and clinical skills and professionalism through exploring lifestyle factors and their influence on health’. “Early clinical experience is devoted to providing teaching and learning opportunities in a wide range of skills essential both to successful completion of the course and developing into a good doctor. These include study, inter-personal communication and clinical skills.”  Programme specification learning outcomes include:  “Demonstrate awareness of the importance of their personal, physical and mental wellbeing and incorporate compassionate self-care into their personal and professional life” “Demonstrate that they can make appropriate clinical judgements when considering or providing compassionate interventions or support for patients who are nearing or at the end of life.” “Be able to communicate effectively, openly and honestly with patients, their relatives, carers…” | No specific information relating to empathy-focused training/learning or learning outcomes focused on empathy | Empathy not specifically mentioned as skill/attribute in selection process | Intranet search – communication research and training in empathy to support palliative care paper identified “VERDIS: Video-based communication research and training in decision-making, empathy and pain management in supportive and palliative care” |
| **University of Oxford Medical Sciences Division**  SEM  GEM | Year 4: ‘main focus is on skills for effective clinical practice…ensure students have acquired sufficient level of skill in…communication…’ ‘integrated with the core rotations are cross-curricular course in communication skills…professionalism..  Year 5: Primary Health Care ‘they learn to communicate.’ Palliative Care ‘Patients from the hospice are invited to talk to the students about their experiences, we have interactive small group lectures on symptom management, and actor-led sessions on communication skills and ethics’ | No specific information relating to empathy-focused training/learning or learning outcomes focused on empathy | Empathy not specifically mentioned as skill/attribute in selection process  Short film of students discussing clinical interview “they [Oxford Medical School] are just trying to tell if you are an empathetic and caring person” | Multiple references to research in empathic healthcare identified through University of Oxford internal search engine. |
| **Plymouth University Peninsula Schools of Medicine and Dentistry**  SEM  MFY | Year 1: Clinical and Communication skills - This module deals with the acquisition of the basic core clinical and communication skills underpinning modern clinical practice.  Year 2: This module deals with the further acquisition of the core clinical and communication skills underpinning modern clinical practice.  Year 3: SSC “… develops the students' written and verbal communication… The module aims to emphasise the development and achievement of skills in: communication…”  MFY: Course aim: “To develop a range of key transferable skills including communication, reflection…”  “Nurturing critical thinking and a caring approach to your medical practice, we’ll help you develop the clinical and communication, teamwork and leadership skills for a top career in medicine.”  “Practise your clinical and communication skills in the safe setting of our Clinical Skills Resource Centre (CSRC), which features specially designed replicas of hospital wards and emergency rooms, with high-specification patient-simulators.”  “Our impressive Clinical Skills Resource Centre (CSRC) allows you to learn vital clinical and communication skills in a safe, supported environment before the pressure is on in a real clinical setting.”  Student experience: “It has been an incredible life experience and it is clear Plymouth and its staff work endlessly to promote compassion, empathy and real-life skills to become top-quality clinicians in the NHS”  “To develop communication skills and team working skills”  Distinctive features: “small group teaching… It provides a protected environment in which to practise clinical and communication skills.” | No specific information relating to empathy-focused training/learning or learning outcomes focused on empathy | MMI ‘we are looking for you to demonstrate these at interview…empathy and being non-judgemental’. | ‘Movecare: Virtual empathic caregivers for the elderly’ project at Centre for Health Technologies |
| **Queens University Belfast School of Medicine**  SEM | “Good communication and examination skills training is undertaken in the Clinical Skills Education Centre with our ‘Patients as Partners’ before students move to actual patient contact in the healthcare environment.”  “Professionalism, ethics, communication, teamwork, and related behavioural science is embedded through the Double-Helical Themes.”  “An extensive suite of on-line clinical and communication skills training resources is also available” | No specific information relating to empathy-focused training/learning or learning outcomes focused on empathy | MMIs designed to test for empathy, amongst other things. | Search of website using term empath - Centre for Medical Education: A new research study by Queen’s University suggests that techniques allowing medical students to ‘walk in the shoes’ of a patient may better prepare them as future doctors. The study involves students wearing temporary ‘cancer tattoo’ transfers for 24 hours after hearing a real story from a patient….”It has improved my empathy towards patients and has given me a much greater respect for what they have to deal with” |
| **University of Sheffield Medical School**  SEM  GEM | “Patients as educators scheme…patients from the local community will volunteer to let us examine them”  Patient-centred approach “students must develop skills and understanding in communication and interpersonal relationships, patients’ perceptions…”  Medicine in Practice Module: “Time spent with GPs within surgeries will also help you to understand a holistic approach to health care as well as building your communication skills and teaching you about medical history taking and examination.”  “with learning about diseases students will learn about the principles and practice of treating patients with drugs, surgery, radiotherapy, psychotherapy and the therapeutic value of good communication”  “Communication skills development including advising patients, breaking bad news and talking with relatives” | No specific information relating to empathy-focused training/learning or learning outcomes focused on empathy | Empathy not specifically mentioned as skill/attribute in selection process | Psychiatry undergraduate teaching lead:  “I am part of a group evaluation of student experience of Balint Groups in undergraduates and their value in developing empathy and compassion.”  “Narrative Masterclass” for medical students and people living with mental health difficulties…short film ‘5 week storying classes’. ‘put medical student in the place of patients’ |
| **University of Southampton School of Medicine**  SEM  GEM  MFY | “The Communication theme runs through all years of medical education incorporating lectures, experiential learning, and, increasingly, working with ‘simulated patients'.”  “A significant part of Communication is the time learners now spend with simulated patients. This increase has come from changing health care policy, curricular initiatives and an increasing emphasis on the assessment of clinical competencies.”  “As part of the Communication theme, medical students are required to attend communication skills workshops, which were developed in order to bring these aspects of the medical curriculum together in a realistic yet ‘safe' environment, drawing on elements of problem-based learning.  A wide variety of scenarios, together with a corresponding set of learning outcomes, were developed. Scenarios were designed to reflect ‘real' situations that medical students may encounter at various stages of their training, rather than situations that they would be unlikely to face until later in their careers.”  Communication is theme during Phase 1  “*From the first year we had contact with patients, which helped develop our communication and patient skills right from the start.*  Medicine in Practice modules include communication skills training | No specific information relating to empathy-focused training/learning or learning outcomes focused on empathy | Empathy not specifically mentioned as skill/attribute in selection process |  |
| **University of St Andrews School of Medicine**  SEM  GEM  MGY  Three years at St Andrews followed by three years at partner schools | GEM: “ScotGEM aims to produce a cohort of high quality, adaptable and compassionate clinical leaders.”  Year 1 – “The complexity and challenge of the cases builds as you and your peers become more effective learners. Case-related clinical and communication skills will be introduced alongside topics such as biochemistry, pharmacology and anatomy.”  “From the outset, the foundations are laid for developing good communication and clinical skills.”  Curriculum addresses core principles including: “Professionalism – development of clinical skills, personal values, interpersonal skills and ethical awareness” and “Reflection – monitoring self-awareness and decision-making through the completion of a portfolio.”  Third year – “provides the opportunity to significantly advance student clinical and communication skills in terms of patient examination techniques and associated procedures  “Clinical skills teaching, including communication skills, takes place in simulated wards and examination and tutorial rooms, supported by video technology. Clinical experience is also offered in the form of clinical placement with patient contact from first year onwards. Professionalism and patient safety are key components of the entire course.”  “Communication skills is an integral part of the curriculum…it’s important for many reasons…better outcomes for patients…”  “safe environments using simulated patients who play the role of a patients to students can practice some of the techniques that we’re discussing with them in an environment where they know there are not going to be any consequences for the real patient…actors are incredibly effective as patients and students are emersed in a room with a simulated patient and they do have a feeling that this patient is experiencing the things that they are talking about” | No specific information relating to empathy-focused training/learning or learning outcomes focused on empathy | Qualities and experience for applying include:  “personal qualities such as empathy, good communication and listening skills, leadership skills and the ability to work in a team” | Programme specification available on website |
| **St George’s, University of London**  SEM  GEM | The professional skills theme equips students with the core patient-centred communication, clinical and procedural skills integral to becoming a doctor. In the early years, clinical and communication skills are developed and integrated through simulated practice involving diverse and authentic clinical scenarios.  ‘Life structure’ module – “Patient examination, clinical and communication skills, history taking and clinical management…are introduced in life structure.”  “Weekly clinical skills and communication skills sessions all occur in the small group format allowing a high degree of learner engagement, role play and reflection.”  Essentials Foundation for Clinical Practice “This three-week module reviews…key clinical skills and procedures required for the workplace. Examples of skills include communication skills sessions…”  Palliative Care “Learning focuses on identifying patients who would benefit from palliative care involvement, communication about end of life care with patients and carers, assessing holistic needs”  General Practice apprenticeship “Starting with an individualised learning needs assessment, this five-week placement uses the breadth of clinical, communication, team-based and ethical learning opportunities” | No specific information relating to empathy-focused training/learning or learning outcomes focused on empathy | MMIs used at selection with scenarios based approach to help “better assess qualities that make a good healthcare professional, such as empathy and respect…” | MOOC (massive open online course) – ‘Developing clinical empathy: Making a difference in Patient Care’. CPD (continuing professional development) Future learn module (open to UG students in healthcare and others)  Centre for Clinical Education - Clinical Communication “We work with students to help you develop empathic practice, so you can develop a relationship of trust with your future patients.”  The Art of Medicine: The Empathy Exams. 2014. Visit by Leslie Jamison to discuss her book and work as an actor teaching medical students empathy |
| **University of Sunderland School of Medicine**  SEM | Years 1 & 2: ‘Overview with early clinical exposure. You will learn the fundamentals…communication skills, basic clinical skills and professionalism.’  “This course will enable you to develop your intellectual, personal and professional capabilities. These attributes include independent thinking, synthesizing information, creative problem solving, communicating clearly, and appreciating the social, environmental and global implications of your studies and activities.”  Phase 2: “Communication skills sessions Experiential learning on clinical placements and other environments” | No specific information relating to empathy-focused training/learning or learning outcomes focused on empathy | “MMIs assess a range of attributes of applicants, including…empathy and insight…” |  |
| **Swansea University Medical School**  GEM | “a strong focus on clinical and communication skills”  Year 1: “Integrated Clinical Method - Half day sessions in each learning week conducted in small groups practicing history taking, communication, examination and practical procedures. Initially lab based using actors, but progressing to real patients and ward based activities across the four years which aim to progressively develop and refine skills.”  Year 2: Doctor as a practitioner 2 “This module enables medical students to further develop their clinical and communication skills… this module enables students to further develop their skills within a safe but clinically-focused environment.”  Year 3 & 4: “ Integrated Clinical Method - Seven half day sessions over the third and fourth years conducted in small groups practicing history taking, communication, examination and practical procedures.”  Year 4: Doctor as a practitioner 4 “This module enables medical students to consolidate their clinical and communication skills within their clinical practice…” | No specific information relating to empathy-focused training/learning or learning outcomes focused on empathy | Empathy not specifically mentioned as skill/attribute in selection process |  |
| **University of Central Lancashire School of Medicine**  SEM (limited no. of UK student places)  MFY (international students only) | “Our degree in medicine (MBBS) will train you to become a professional, compassionate doctor…considerable emphasis on developing your expertise in a whole range of practical areas, including clinical, communication, observation, teamwork and leadership skills.  Across all years: “develop your initial skills to communicate effectively with patients, carers and healthcare professionals”  “Three course themes which run throughout the five-year programme...Medical Skills and Quality Care (MSQC) – develop the clinical and communication skills needed by a medical doctor, practising skills in a laboratory setting and with patients.”  Year 1 & 2: “…begin to develop your professional and clinical skills. This will mean weekly communication and clinical sessions with members of staff and simulated scenarios with actors. We encourage you to develop a reflective and enquiring approach…” | No specific information relating to empathy-focused training/learning or learning outcomes focused on empathy | “At interview, assessors will score the candidate for the following broad criteria… Professional attitudes and values (flexibility, integrity, ethics, empathy, honesty and conscientiousness)” |  |
| **University College London Medical School**  SEM | “Clinical and Professional Practice (CPP) modules run ‘vertically’ through the entire MBBS programme and included…Communication and Patient Safety/Experience”  CPP “Effective communication. Recognise workplace based teaching around real patient encounters as a process of integrative thinking… Develop core skills and an orientation for effective communication, ethical practice, and working within the framework of the law and professional guidance.”  CPP module “Examine the co-ordinated efforts to prevent harm to patients within healthcare. Through openness and transparency, communication and empathy, we aim to deliver a safe, patient-centred clinician.”  SSCs in years one, two and six allow students to pursue special interests…include choices in…the arts and humanities…” | No specific information relating to empathy-focused training/learning or learning outcomes focused on empathy | “Interviewers score the candidates on the following qualities… Professional attitudes and values (factors such as flexibility, integrity, empathy, honesty, conscientiousness and compassion)” |  |
| **University of Warwick Medical School**  GEM | Year 2: Advanced Cases 1 “During the block you will develop your skills in critical analysis of clinical information, team working and communication and start to apply a problem based approach to your history taking and examination.”  “Case-Based Learning (CBL) is at the core of the MB ChB curriculum and is integrated across all four years of the programme…Developing team working, communication and professional skills”  SSC options include ‘Mindfulness in Clinical Practice’ ‘An introduction to Medical Humanities’ ‘the Medical Mind in Literature and Culture’. | No specific information relating to empathy-focused training/learning or learning outcomes focused on empathy | MMIs ‘the values and competencies we look for are…empathy’. | “The Patient Journey: This is a project co-designed during a design thinking workshop by patients, staff and students. Students follow a patient from home, to outpatients and back again. Student and patient interviews of their experiences will be analysed to see if this could become part of the curriculum to improve empathy.”(2020) |
| **Ulster University, School of Medicine**  GEM | “The vision for the medical school is to… Produce doctors able to deliver whole person care with skill and compassion, as members and leaders of diverse clinical teams, in partnership with patients and clients.”  “Our clinical and communication skills tutors are experienced local GPs sharing their real-life experiences with you” | No specific information relating to empathy-focused training/learning or learning outcomes focused on empathy | “Each MMI followed a specific blueprint and all the questions mapped to this consistently…empathy” |  |

*Description is not exact or exhaustive of course overview/curriculum/teaching/learning activities but is a brief summary of available online information

**Standard Entry Medicine SEM, Graduate Entry Medicine GEM, Medicine with a Foundation Year MFY, Medicine with a Gateway Year MGY
